# A high-resolution map of reactive nitrogen inputs to China

**DOI:** 10.1038/s41597-020-00718-5

**Published:** 2020-11-11

**Authors:** Sitong Wang, Xiuming Zhang, Chen Wang, Xiuying Zhang, Stefan Reis, Jianming Xu, Baojing Gu

**Affiliations:** 1 College of Environmental and Resource Sciences, Zhejiang University, Hangzhou, 310058 P.R. China; 2 School of Agriculture and Food, The University of Melbourne, Melbourne, Victoria 3010 Australia; 3 International Institute for Earth System Science, Nanjing University, Nanjing, 210023 P.R. China; 4 UK Centre for Ecology & Hydrology, Bush Estate, Penicuik, Midlothian EH26 0QB UK; 5 University of Exeter Medical School, European Centre for Environment and Health, Knowledge Spa, Truro, TR1 3HD UK; 6 Zhejiang Provincial Key Laboratory of Agricultural Resources and Environment, Zhejiang University, Hangzhou, 310058 China

**Keywords:** Element cycles, Element cycles

## Abstract

To feed an increasingly affluent population, reactive nitrogen (Nr) inputs to China’s lands and waters have substantially increased over the past century. Today, China’s Nr emissions account for over one third of global total emissions, leading to serious environmental pollution and health damages. Quantifying the spatial variability of Nr inputs is crucial for the identification of intervention points to mitigate Nr pollution, which, however, is not well known. Here, we present a database describing Nr inputs to China for the year 2017 with a 1 km × 1 km resolution, considering land use and Nr sources, compiled by using the CHANS model. Results show that the North China Plain, the Sichuan Basin and the Middle-Lower Yangtze River Plain are hotspots of Nr inputs, where per hectare Nr input is an order of magnitude higher than that in other regions. Cropland and surface water bodies receive much higher Nr inputs than other land use types. This unique database will provide basic data for research on environmental health and global change modelling.

## Abstract (summary)

**Table:** 

Measurement(s)	reactive nitrogen species
Technology Type(s)	computational modeling technique
Factor Type(s)	land use • geographic location • reactive nitrogen source
Sample Characteristic - Location	China

Machine-accessible metadata file describing the reported data: 10.6084/m9.figshare.12971939

## Background & Summary

China produced the world’s largest amount of reactive nitrogen (Nr) through Haber–Bosch nitrogen (N) fixation (HBNF), around 40 Tg Nr in 2017^1^. A substantial input of Nr from agricultural and other activities has resulted in a range of adverse effects on human health and environmental quality, including the loss of biodiversity, soil acidification, and eutrophication^2^. Although early successes in mitigating Nr pollution have been observed in recent years with the introduction of strict pollution control measures, Nr pollution still presents an area of widespread concern in China^3^. To better understand the pathways for Nr losses and identify mitigation options, it is crucial to assess the current status and characteristics of N inputs to China’s lands and water bodies. Previous studies on Nr inputs to China typically have a spatial resolution down to province or county level. However, this is not sufficient to use for a detailed estimation on health or environmental effects derived from Nr uses^4,5^. Meanwhile, more and more studies utilize complex process-based simulation models requiring high resolution gridded data, but so far lack high-resolution maps of Nr input^6^.

Crop production has markedly increased over the past 40 years in China, due to the substantial increase in the use of synthetic N fertilizers^7^. In 2017, the total N fertilizers applied to Chinese croplands amounted to 29 Tg N (173 kg/ha for rice, 213 kg/ha for wheat and 183 kg/ha for maize), accounting for some 30% of global total fertilizer use on only 9% of global cropland, while producing far lower crop yields compared to the global average^8^. Meanwhile, China raises around 40% of global livestock, and manure N has become an important source of N inputs to lands and water bodies. These features lead to a much higher Nr loading in China compared to other global regions. However, due to the lack of a comprehensive, high resolution dataset on livestock distribution, spatially allocating these Nr inputs on different land areas and catchments is a grand challenge. Furthermore, livestock distribution also changes annually with market and policy regulations, such as the relocating pigs program^9^, adding complications to the compilation of robust spatial datasets. Other than agricultural sources of Nr inputs, nitrogen oxide (NOx) emissions from fossil fuel combustion also contribute to Nr deposition to land and water. For instance, the Nr deposition can contribute to over 30% of total Nr input to the Lake Tai in East China^10^, and NOx has a large share in the Nr deposition.

Previous studies have quantified the spatial distribution of some of these Nr fluxes, such as emissions from fertilizer use^11^, or atmospheric Nr deposition, separately. However, there are few studies which integrate all relevant N inputs with high spatial resolution to allow for an identification of the key drivers of adverse effects on human environmental health. To fill this knowledge gap and at the same time update the quantification of N fluxes to the most recent year, 2017, we calculated the overall N budget for China first using the Coupled Human And Natural System (CHANS) model (https://person.zju.edu.cn/en/bjgu#930811). CHANS includes 14 different subsystems covering all the natural and anthropogenic sources of Nr input, recycling and losses. Then, we extract all the N input fluxes to land and water bodies, estimate their spatial distribution with a spatial resolution of 1 km × 1 km using spatial indicators such as land use or population distribution. This results in a unique database which will help users to explore spatial patterns of Nr inputs in China, to assess N input on sensitive ecosystems within safe boundary and support the development of mitigation strategies for Nr pollution in China.

## Methods

### Database structure

**Fig. 1:**
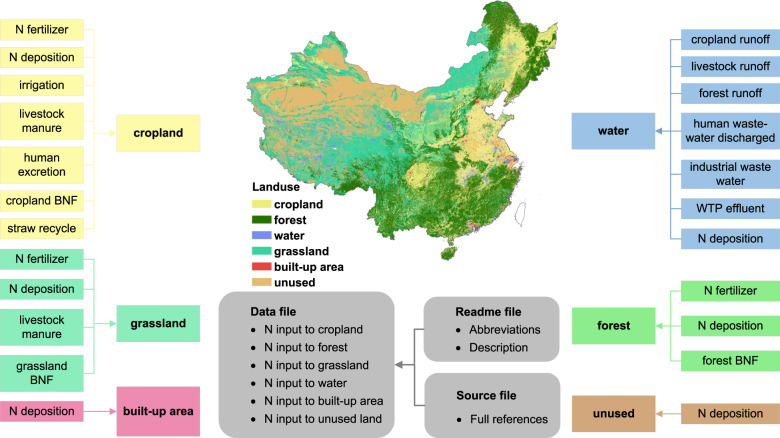
A general view of N inputs to 6 land-use types in China and the framework of The Coupled Human And Natural Systems Nitrogen Cycling Model Spatial Distribution (CHANS-SD) 1.0 database. N input to cropland comprises of N fertilizer, N deposition, irrigation, livestock manure, human excretion, cropland BNF (Biological N Fixation), and straw recycle; N input to forest comprises of N fertilizer, N deposition and forest BNF; N input to grassland comprises of N fertilizer, N deposition and grassland BNF; N input to water comprises of cropland runoff, livestock runoff, forest runoff, human wastewater discharged, industrial wastewater, WTP (Water Treatment Plant) effluent and N deposition; N input to built-up area and unused land is N deposition. The data of Taiwan is absent. The data consists of three files: the ‘data file’ (CHANS-SD 1.0 Data File) is the main file, includes N inputs in all 6 land-use types. The ‘readme file’ (CHANS-SD 1.0 Read Me) explains the abbreviations and units, and the ‘source file’ includes the full references used in the database. Base map is applied without endorsement from GADM data (https://gadm.org/).

The Coupled Human And Natural Systems Nitrogen Cycling Model Spatial Distribution (CHANS-SD) 1.0 database consists of three files (Fig. 1). The ‘data file’ provides N inputs of 6 land use types, including cropland, forest, grassland, water, built-up area and unused land. The ‘readme file’ explains the abbreviations used in the ‘data file’ and ‘source file’, and provides the units of all variables (Fig. 1). The ‘source file’ includes the full references and input data used in the database (Fig. 1).

### Data compilation

We applied the CHANS model to calculate N inputs by land use type. The CHANS model calculates all nitrogen (N) fluxes that can be identified, together with the linkages among subsystems, within a country, state (province), city or watershed, with a mass balance principle. The system is divided into 14 subsystems: cropland, grassland, forest, livestock, aquaculture, industry, human, pet, urban green land, wastewater treatment, garbage treatment, atmosphere, surface water, and groundwater. N cycling starts from the entry of reactive N (Nr) that activated from N_2_ into the system or from Nr direct input to the system from outside, and terminates when Nr is transformed to N_2_ or lost to outside the system.

**Fig. 2:**
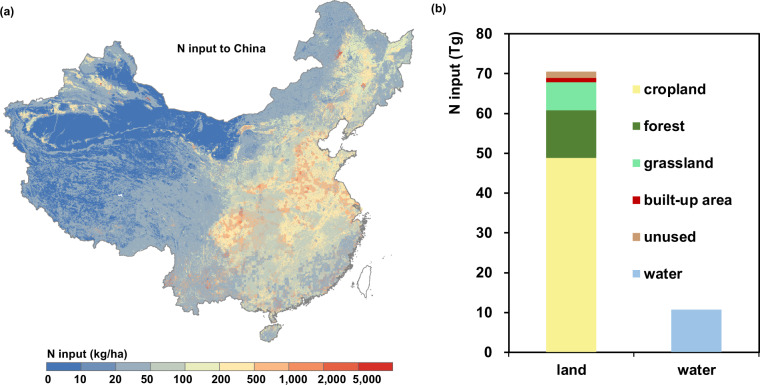
N input to China and proportion of its six components. (**a**) A spatial distribution map of N input to China, N input in east of China is much higher than that in west of China; North Plain, Sichuan Basin and the North East Plain currently receive the highest N loads (The data of Taiwan is absent). (**b**) Proportion of N input to cropland, forest, grassland, built-up area, unused land and water, respectively; proportion of N input to cropland is highest among all land-use types. Base map is applied without endorsement from GADM data (https://gadm.org/).

N input to cropland subsystem is the largest component of N input to China, including N fertilizer, atmospheric N deposition, N from irrigation, N from livestock manure, N from human excretion, cropland biological N fixation (BNF) and straw recycle. N input to forest subsystem, including forest BNF and N deposition, is the second largest component of N input to China given the large area of forest. N input into surface water subsystem, including Nr runoff from cropland, livestock and forest, human wastewater discharged, industrial waste water, WTP (Water Treatment Plant) effluent and N deposition, is the third largest component of N input in China (Fig. 2). The details of the CHANS model including all the code and parameters of N cycling, and the protocols for the calculation of all related N fluxes can be found from https://person.zju.edu.cn/en/bjgu#930811.

The National Bureau of Statistics of China^1^ provides data of cropland N fertilizer use, planting area, livestock and population in the statistical yearbook of each province. Taiwan, Hong Kong, and Macao were not included owing to data limitations. Gridded datasets of land use and GDP is derived from the Resource and Environment Data Cloud Platform^12^, and data on China’s hydro-basin distribution is collected from the FAO website GeoNetwork^13^.

**Fig. 3:**
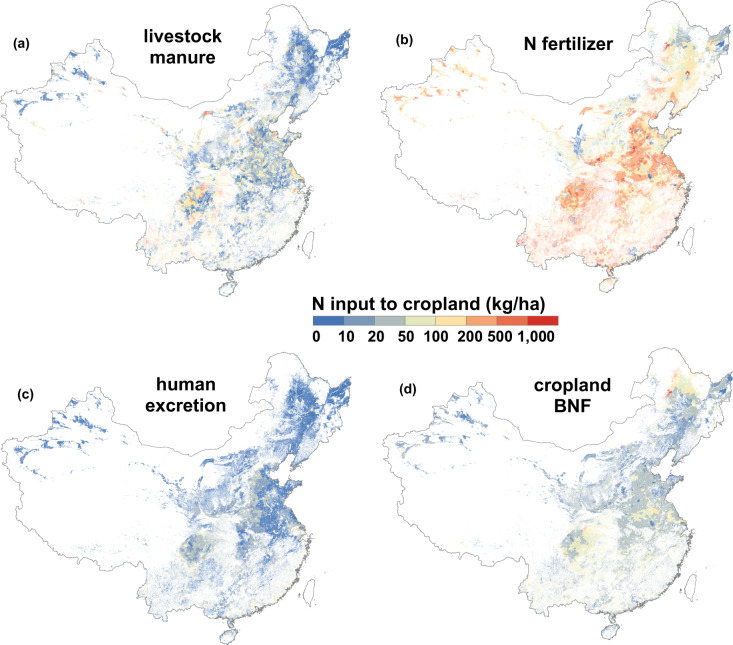
Important components of N inputs to cropland. (**a**), N input from livestock manure to cropland, high values (>100 kg/ha) concentrate in the North Plain; (**b**), N input from N fertilizer to cropland, compared to other items, high values (>100 kg/ha) are widely distributed across China; (**c**), N input from human excretion to cropland; (**d**), N input from cropland BNF (biological N fixation) to cropland. The data of Taiwan is absent. Base map is applied without endorsement from GADM data (https://gadm.org/).

In the cropland subsystem, livestock manure input to cropland (*MANURE*_*an*_, kg/yr) was calculated based on animal population (*POP*_*an*_, head), excretion factor (*EXCRE*_*an*_, kg N/head/yr) and rate of livestock manure applied to cropland (*RE*_*an*_, %) according to Eq. (1) (Fig. 3a),

1 $${MANURE}_{an}={POP}_{an}\times {EXCRE}_{an}\times {RE}_{an}$$

Amounts of chemical N fertilizer consumption at county scale were obtained from the statistical yearbook of counties (Fig. 3b). Human manure to cropland (*MANURE*_*hu*_, kg /yr) was calculated by urban population (*POP*_*ur*_, person), rural population (*POP*_*ru*_, person), excretion factor (*EXCRE*_*hu*_, kg N/person/yr), rate of urban excretion return to cropland (*RE*_*ur*_, %) and rate of rural excretion return to cropland (*RE*_*ru*_, %) according to Eq. (2) (Fig. 3c).

2 $${MANURE}_{hu}=({POP}_{ur}\times {RE}_{ur}+{POP}_{ru}\times {RE}_{ru})\times {EXCRE}_{hu}$$

Cropland BNF (*CBNF*, kg/yr) was calculated by planting area (*area*, ha) and N fixation rate (*r*_*fix*_, kg N/ha/yr) according to Eq. (3) (Fig. 3d),

3 $$CBNF=area\times {r}_{fix}$$

Straw recycling and N input from irrigation are calculated by using nationally uniform values (Table 1).

**Table 1:** A summary of N inputs to six land-use types in 2017 in China.

Land-use type	Item	N input (Tg)
Cropland	N fertilizer	28.9
	N deposition	5.9
	irrigation	0.7
	livestock manure	5.3
	human excretion	1.6
	cropland BNF	4.6
	straw recycle	2.4
Forest	N fertilizer	2.4
	N deposition	5.5
	forest BNF	4.1
Grassland	N fertilizer	0.6
	grassland BNF	3.0
	N deposition	3.5
	irrigation for artificial grassland	0.0
	livestock manure	3.7
Water	cropland runoff	2.4
	livestock runoff	1.0
	forest runoff	1.3
	human wastewater discharged	1.9
	industrial wastewater	2.3
	WTP effluent	1.2
	N deposition	0.6
Built-up area	N deposition	1.0
Unused land	N deposition	1.6

Note, cropland and surface water receive much higher Nr inputs than other land use types, and approximately half of the national inputs are from agricultural activities.

N input to the grassland subsystem include N fertilizer, manure recycle, N deposition, grassland BNF and irrigation for artificial grassland. N input in forest subsystem include N fertilizer to artificial forests, N deposition and forest BNF.

**Fig. 4:**
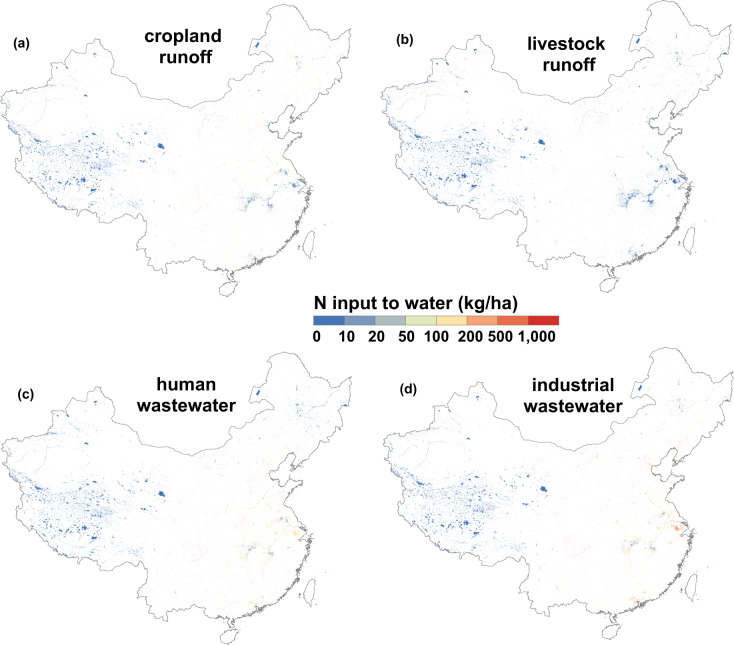
Important components of N inputs to water. (**a**) N input from cropland runoff to water, shows similar distribution with N input to cropland; (**b**) N input from livestock runoff to water, shows similar distribution with N input from livestock manure to cropland; (**c**) N input from human wastewater to water, shows similar distribution with population; (**d**) N input from industrial wastewater to water, shows similar distribution with GDP. The data of Taiwan is absent. Base map is applied without endorsement from GADM data (https://gadm.org/).

In the surface water system, N inputs to each watershed include runoff from cropland, livestock, and forest areas, human wastewater discharge, industrial wastewater, WTP effluent and atmospheric N deposition. Cropland runoff was calculated by spatial distribution of cropland input (Fig. 4a). Livestock runoff using the spatial distribution of livestock manure (Fig. 4b). Finally, forest runoff was calculated by applying the spatial distribution of forest areas. Human wastewater discharge was calculated based on the spatial distribution of the human population (including urban and rural populations) (Fig. 4c). Industrial wastewater was calculated by using the spatial distribution of GDP since industrial output is highly correlated with GDP on regional scale (Fig. 4d). Similarly, the distribution of WTP and its effluent Nr are highly correlated with urban population on regional scale, therefore, WTP effluent was calculated by spatial distribution of urban population.

For N deposition, the satellite observations on NO_2_ columns were derived from GOME-2. GOME-2 overpass times provided global coverage of NO_2_ with a variable ground spatial resolution of 80 km × 40 km (every day). We used the monthly TEMIS NO_2_ product at a spatial resolution of 0.25° latitude × 0.25° longitude downloaded from the website of Tropospheric Emission Monitoring Internet Service^14^. The satellite observations on NH_3_ columns were derived from IASI onboard the meteorological platforms MetOp-A and MetOp-B^15^ with an elliptical footprint of 12 × 12 km up to 20 × 39 km depending on the satellite-viewing angle. The daily NH_3_ columns were downloaded from IASI Portal^16^. We processed the daily data into the monthly NH_3_ columns averaged by daily observations at a horizontal resolution of 0.25° latitude × 0.25° longitude using the arithmetic mean method^17,18^. N deposition of every subsystem was obtained from the national N deposition spatial distribution map by ‘spatial join’ tool of ArcGIS 10.6.

## Data Records

The data are available in a single dataset^19^, which consists of three files: the ‘data file’ (CHANS-SD 1.0 Data File) is the main file, includes N inputs in all 6 land-use types. The ‘readme file’ (CHANS-SD 1.0 Read Me) explains the abbreviations and units, and the ‘source file’ includes the full references used in the database (Fig. 1). The CHANS-SD 1.0 database is the most comprehensive and up-to-date measurement-based dataset of Nr inputs over different land-use types (e.g., cropland, forest, grassland, built-up area and unused land) in China.

## Technical Validation

All data included in the CHANS-SD 1.0 database are derived from published statistical yearbook data of 293 prefecture-level cities, including 2,311 counties, and presents the best available dataset for China in 2017. Thorough quality assurance and control (QA/QC) has been conducted with each data record having been checked for possible errors and the extreme values were excluded. The data of N input to cropland system (e.g. N fertilizer) is less uncertain than the data of N input to water system, since N input to cropland is calculated directly from governmental statistical data while N input to water is calculated from data of other subsystems (e.g., cropland, livestock, forest, human, wastewater and industry). The stability of CHANS model has been validated by international peers and we have published many papers using the data and methods of this model^6,20–22^, and the data of CHANS model show similar spatial distribution with other studies^23,24^. Overall, CHANS-SD 1.0 database provides high-quality open-access information on N input to China’s land and water.

## Data Availability

No custom computer code was used to generate the data described in the manuscript. A CHANS Excel Calculator describes the data we used is available in figshare data record (10.6084/m9.figshare.12637391.v5).
